# The vascular access questionnaire: a single centre UK experience

**DOI:** 10.1186/s12882-019-1493-9

**Published:** 2019-08-05

**Authors:** M. Field, A.Z Khawaja, J. Ellis, T. Nieto, J. Hodson, N. Inston

**Affiliations:** 1 Department of Renal Transplantation and Vascular Access Surgery, Queen Elizabeth Hospital, Edgbaston, Birmingham, B15 2TH UK; 2 Institute of Translational Medicine, Queen Elizabeth Hospital, Edgbaston, Birmingham, UK

**Keywords:** Vascular access, Patient reported outcomes, Vascular access questionnaire, Patient experience

## Abstract

**Background:**

Haemodialysis is capable of prolonging life in patients with end stage renal disease, however this therapy comes with significant negative impact on quality of life. For patients requiring haemodialysis, the need for an adequately functioning vascular access (VA) is an everyday concern. The Vascular Access Questionnaire (VAQ) provides a mechanism for identifying and scoring factors in haemodialysis that impact on patients’ quality of life and perception of their therapy.

**Methods:**

Between April 2017–18 the VAQ was administered to prevalent haemodialysis patients at 10 units in the West Midlands via structured interviews.

**Results:**

749 of 920 potentially eligible patients completed the survey. The mean VAQ score was seen to improve significantly with age (7.7 in < 55 vs. 3.8 in 75+) and the duration of access (8.9 if less than 1 month old vs. 5.0 at a year). Better average scores were demonstrated for Arteriovenous fistulas (AVF) than other modalities (AVF 5.1 vs. AVG (arteriovenous grafts) 7.2 vs. CVC (central venous catheter) 6.6). There was no significant difference in scores between fistulas on non-dominant or dominant arms, with both having a mean of 5.2 (*p* = 0.341).

**Conclusions:**

Overall, better satisfaction scores were seen in AVF. The presence of an AVF on the non-dominant arm was not a concern for the majority of patients and did not affect the VAQ score. A number of factors were identified that can influence VAQ satisfaction score.

**Electronic supplementary material:**

The online version of this article (10.1186/s12882-019-1493-9) contains supplementary material, which is available to authorized users.

## Background

Guidelines, best practice policies and quality improvement initiatives support a hierarchy of arteriovenous fistulas (AVFs), arteriovenous grafts (AVGs) and central venous catheters (CVCs) as a last choice, with regards to permanent form of Vascular Access (VA) [1]. These recommendations are based on associated clinical outcomes derived from population studies. These so called “hard outcome measures” are increasingly recognized as deficient in factors important to patients, and may not be the only way of comparing different options for the patient [2].

For patients with chronic kidney disease, multiple aspects of care can impact on quality of life [3]. Haemodialysis patients consistently have lower Quality of Life (QoL) scores than patients who are pre-dialysis or have a transplant [4]. Previous studies demonstrated a negative impact of a CVCs [5, 6]. However, these studies only reported small difference in Health Related Quality of Life (HRQL) between different access modalities, despite there being differences in the reported Vascular Access Questionnaire scores (VAQ) [7] .

A patient’s vascular access is a visible reminder of their reliance on a dialysis and that they have a life threatening disease. All forms of VA require maintenance and are associated with complications and can impact on the patients overall quality of life [8].

The patient experience or satisfaction with their vascular access may play an important role in their choices for vascular access [9]. Previous studies have suggested that patients are concerned with the physical aspects of AVFs, particularly their appearance, associated pain and bleeding. The choice between the pain of having an AVF cannulated versus the needle free benefit of a CVC are a striking example. Previous work has suggested that these concerns are more important to patients and outweigh the potential benefits of an AVF, such as lower infection rate [10, 11].

Defining and a better understanding of patient’s views and experiences is fundamental to tailoring individualised care, addressing concerns to improve AVF uptake and quality of care of the patient as a whole.

The Vascular Access Questionnaire (VAQ) was described in 2008 by Quinn et al. and consists of a patient-reported questionnaire composed of 17 vascular access related questions (Additional file 1: Table S1), with responses on a five-point Likert scale which are summed, to give a Vascular Access Score, a lower overall score indicating greater satisfaction. Quinn et al. analysed responses from 222 patients and, although no statistically significant differences were revealed between AVF and CVC scores, patients with AVFs tended to be more concerned by physical symptoms (pain, bleeding, bruising) than patients with a CVC [11, 12].

The aim was to apply the VAQ in a regional, multi-ethnic dialysis population, to identify patient characteristics that influence their perception and outcomes of VA, with the aim of defining areas for quality improvement initiatives, including improvement of the information and service our patients receive.

## Methods

Between April 2017 and April 2018, the VAQ was administered to in-centre patients established on haemodialysis at ten dialysis units within a UK region (West Midlands) served by the Queen Elizabeth Hospital, Birmingham. All patients undergoing haemodialysis in the chronic centres who were able to consent were approached. Patients undergoing dialysis on the acute unit or home haemodialysis were excluded.

A data collection instrument collected responses to the VAQ, demographics, comorbidities, vascular access history and interventions, and treatment satisfaction. Questions gauged the patient’s perception of the dialysis nurse’s attitude to their access and their view on the best access option for their health. In addition, three open ended, free response questions were included to assess:

1. whether patients felt they had received sufficient access information prior to starting dialysis
2. reasons for not considering an AVF in patients with a CVC
3. issues with AVF on the patient’s dominant arm.

The interview was conducted in the patients preferred language. Non-participation reason data was also recorded.

Data was managed using REDCap electronic data capture instrument hosted at the University of Birmingham [13] and exported for analysis to IBM SPSS 22 (IBM Corp. Armonk, NY), and Prism version 7.0. Institutional audit approval was granted (CARMS-12695).

### Statistical methods

VAQ scores had a highly skewed distribution, and so were reported as medians and interquartile ranges (IQRs). However, since the VAQ score produced discrete values, this approach lacked the granularity to clearly demonstrate differences between groups. As a result, the scores were also summarised using means. Comparisons across nominal factors were performed using Mann-Whitney or Kruskal-Wallis groups where there were two or more than two groups, respectively. Significant Kruskal-Wallis tests were followed by post-hoc pairwise comparisons using Dunn’s test. For ordinal and continuous factors, Spearman’s rho correlations were used to assess the significance of any associations. A multivariable analysis was then performed, to identify independent predictors of the VAQ score. Due to the distribution of the score, it was not possible to produce a reliable linear regression model. Instead, the scores were dichotomised, based on the upper quartile of the distribution, with the resulting variable set as the dependent variable in a binary logistic regression model.

Subgroup analyses were then performed, to assess the differences between the scores for the individual component questions of the VAQ score across a range of demographic and access-related factors. All analyses were performed using IBM SPSS 22 (IBM Corp. Armonk, NY), with *p* < 0.05 deemed to be indicative of statistical significance throughout.

## Results

### Demographics

Nine hundred twenty patients were identified and 749 patients (81.4%) completed the survey. Of those who did not complete the survey, 64.3% (*n* = 110) were not present at the time of the visit to their dialysis unit, 16.4% (*n* = 28) did not want to participate and 19.3% (*n* = 33) could not consent.

The 749 patients included had a median age of 65 years (IQR: 55–76), 57.8% were male and 49.3% White ethnicity. The median length of haemodialysis was 3 years (IQR: 2–7) and the majority had an AVF (72.0%) as their mode of vascular access. Further details in Tables 1, 2, 3 and 4.

**Table 1:** Patient Demographics

	Total N		VAQ Score		
		N (%)	*Mean*	*Median (IQR)*	*p-Value*
Age (Years)	749				<0.001*
*<55*		175 (23.4%)	7.7	6 (2–11)	
*55–64*		183 (24.4%)	6.2	4 (1–8)	
*65–74*		178 (23.8%)	4.9	3 (1–7)	
*75+*		213 (28.4%)	3.8	3 (1–6)	
Gender	749				**<0.001**
*Female*		316 (42.2%)	6.7	5 (2–10)	
*Male*		433 (57.8%)	4.7	3 (1–7)	
Ethnicity	748				**<0.001**
*White*		369 (49.3%)	5.5	4 (2–8)	
*Asian*		252 (33.7%)	5.2	3 (0–7)	
*Black*		124 (16.6%)	6.4	5 (2–10)	
*Mixed*		3 (0.4%)	4.7	3 (3–8)	
Peripheral Vascular Disease	749				**0.011**
*No*		641 (85.6%)	5.3	3 (1–8)	
*Yes*		108 (14.4%)	7.2	5 (2–10)	
Cardiac disease	749				0.055
*No*		503 (67.2%)	5.2	3 (1–7)	
*Yes*		246 (32.8%)	6.2	4 (1–9)	
Diabetes	749				0.195
*No*		450 (60.1%)	5.8	4 (1–9)	
*Diet Controlled*		66 (8.8%)	4.2	3 (1–6)	
*Tablet Controlled*		42 (5.6%)	4.0	3 (0–7)	
*Insulin*		191 (25.5%)	5.8	4 (1–8)	
Unit	749				**<0.001**
*Unit 1*		43 (5.7%)	3.6	2 (0–4)	
*Unit 2*		90 (12.0%)	4.5	3 (1–7)	
*Unit 3*		116 (15.5%)	4.6	3 (0–7)	
*Unit 4*		68 (9.1%)	5.1	4 (2–8)	
*Unit 5*		79 (10.5%)	5.3	3 (1–7)	
*Unit 6*		72 (9.6%)	5.3	3 (1–7)	
*Unit 7*		96 (12.8%)	6.1	5 (2–8)	
*Unit 8*		75 (10.0%)	6.3	4 (1–9)	
*Unit 9*		56 (7.5%)	7.8	7 (2–12)	
*Unit 10*		54 (7.2%)	7.6	7 (4–12)	
Years of Haemodiaysis	748				0.235*
*<2*		188 (25.1%)	5.1	3 (1–8)	
*2–3*		197 (26.3%)	5.2	3 (1–7)	
*4–7*		188 (25.1%)	6.0	4 (2–9)	
*8+*		175 (23.4%)	5.7	4 (1–8)	

*p*-Values are from Mann-Whitney or Kruskal-Wallis tests, unless stated otherwise, and bold *p*-values are significant at *p* < 0.05

*p-Value from Spearman’s rho, as the factor is ordinal

**Table 2:** Current access

	Total		VAQ Score		
	N	N (%)	*Mean*	*Median (IQR)*	*p*-Value
Current Mode of Vascular Access	749				**0.004**
*AVF*		539 (72.0%)	5.1	3 (1–7)	
*AV graft*		34 (4.5%)	7.2	5 (3–10)	
*CVC*		174 (23.2%)	6.6	5 (1–9)	
*CVC and Fistula*		2 (0.3%)	10.0	10 (10–10)	
Duration of Current Access?	748				0.003*
*<1 month*		19 (2.5%)	8.9	8 (4–12)	
*1 month - 6 months*		101 (13.5%)	6.2	4 (2–9)	
*6 months − 12 months*		98 (13.1%)	7.1	5 (1–11)	
*over 1 year*		530 (70.9%)	5.0	3 (1–7)	
Current Fistula**	538				0.229
*Brachiobasilic*		53 (9.9%)	5.6	3 (2–8)	
*Brachiocephalic*		273 (50.7%)	5.2	3 (1–7)	
*Radiocephalic*		212 (39.4%)	4.7	3 (1–7)	
Current Graft**	34				0.090
*Lower arm*		2 (5.9%)	1.5	2 (0–3)	
*Upper arm*		27 (79.4%)	7.0	5 (2–9)	
*Upper leg*		5 (14.7%)	10.4	12 (10–13)	
Current CVC**	174				0.120
*Femoral*		15 (8.6%)	10.9	10 (2–19)	
*Jugular*		154 (88.5%)	6.3	5 (2–9)	
*Other*		5 (2.9%)	6.4	7 (1–8)	
Fistula/Graft on Dominant Arm***	570				0.341
*No*		445 (78.1%)	5.2	3 (1–7)	
*Yes*		125 (21.9%)	5.2	3 (2–7)	
Does This Cause Issues?****	125				**0.002**
*No*		88 (70.4%)	4.3	3 (1–7)	
*Yes*		37 (29.6%)	7.4	6 (3–8)	

*p*-Values are from Mann-Whitney or Kruskal-Wallis tests, unless stated otherwise, and bold *p*-values are significant at *p* < 0.05

*p-Value from Spearman’s rho, as the factor is ordinal

**For the subgroup of patients with the stated access type

***Excludes *N* = 5 with leg grafts

****Does having the fistula/graft on the dominant arm cause issues, in those where this question was applicable

**Table 3:** Previous access

	Total		VAQ Score		*p-Value*
	N	N (%)	*Mean*	*Median (IQR)*	
Any Previous Access**	749				**<0.001**
*No*		601 (80.2%)	5.0	3 (1–7)	
*Yes*		148 (19.8%)	7.8	6 (2–12)	
Previous CVC**	749				**<0.001**
*No*		644 (86.0%)	5.2	3 (1–7)	
*Yes*		105 (14.0%)	7.9	6 (3–12)	
Previous Fistula**	749				0.903
*No*		712 (95.1%)	5.5	4 (1–8)	
*Yes*		37 (4.9%)	6.9	4 (1–7)	
Previous Graft**	749				0.194
*No*		737 (98.4%)	5.5	4 (1–8)	
*Yes*		12 (1.6%)	8.3	7 (2–13)	
Number of Previous CVC	748				<0.001*
*0*		244 (32.6%)	4.7	3 (1–7)	
*1 to 5*		437 (58.4%)	5.4	4 (1–8)	
*6 to 10*		47 (6.3%)	9.6	7 (3–14)	
*>10*		20 (2.7%)	9.8	8 (3–18)	
Radiological Intervention					
On Graft/Fistula***	575				**<0.001**
*No*		428 (74.4%)	4.6	3 (1–7)	
*Yes*		147 (25.6%)	7.0	5 (2–10)	
On CVC***	175				0.760
*No*		139 (79.4%)	6.5	5 (2–9)	
*Yes*		36 (20.6%)	7.6	6 (1–11)	

*p*-Values are from Mann-Whitney or Kruskal-Wallis tests, unless stated otherwise, and bold *p*-values are significant at *p* < 0.05

*p-Value from Spearman’s rho, as the factor is ordinal

**Previous access in the year prior to questioning. Categories are not mutually exclusive – respondents were asked to tick all that applied

***In the last year, in those patients who had been treated with the stated access type

**Table 4:** Satisfaction with Treatment

	Total		VAQ Score		*p-Value*
	N	N (%)	*Mean*	*Median (IQR)*	
Satisfaction with Current Access	748				<0.001*
*Very Dissatisfied*		4 (0.5%)	8.0	8 (5–11)	
*Somewhat Dissatisfied*		17 (2.3%)	11.1	9 (6–15)	
*Somewhat Satisfied*		83 (11.1%)	10.3	8 (5–15)	
*Very Satisfied*		644 (86.1%)	4.8	3 (1–7)	
Recommend Current Access	748				<0.001*
*No*		37 (4.9%)	8.6	6 (4–12)	
*Maybe Not*		29 (3.9%)	7.9	9 (3–11)	
*Maybe*		59 (7.9%)	7.5	6 (2–12)	
*Yes*		623 (83.3%)	5.1	3 (1–7)	
Access Easy to Use?	747				<0.001*
*Very Difficult*		3 (0.4%)	22.0	19 (7–40)	
*Somewhat Difficult*		23 (3.1%)	11.2	9 (6–15)	
*Somewhat Easy*		112 (15.0%)	7.1	6 (2–9)	
*Very Easy*		609 (81.5%)	5.0	3 (1–7)	
Which do Nurses Prefer?	744				0.352
*AVF*		270 (36.3%)	5.5	3 (1–7)	
*Equally Happy*		177 (23.8%)	5.7	4 (1–8)	
*CVC*		71 (9.5%)	5.8	5 (2–9)	
*Not Sure*		226 (30.4%)	5.4	4 (1–8)	
Which is Better for Your Health?	748				0.414
*AVF*		553 (73.9%)	5.7	4 (1–8)	
*No Difference*		39 (5.2%)	4.7	4 (0–7)	
*CVC*		52 (7.0%)	4.4	3 (1–6)	
*Not Sure*		104 (13.9%)	5.3	4 (2–7)	

*p*-Values are from Mann-Whitney or Kruskal-Wallis tests, unless stated otherwise, and bold *p*-values are significant at *p* < 0.05

*p-Value from Spearman’s rho, as the factor is ordinal

### Factors associated with the VAQ score

The VAQ score followed a skewed distribution, with a mean of 5.5 and a median of 4 (IQR: 1–8). The associations between the score and a range of demographic factors are reported in Table 1. The VAQ score was found to improve significantly (lower scores) with age (*p* < 0.001), from a mean of 7.7 in those aged < 55 years to 3.8 in those that were 75+ years, and to be significantly worse (higher scores) in females (mean: 6.7 vs. 4.7 in males, p < 0.001). A significant difference between ethnicities was detected (*p* = 0.001), with post-hoc analysis finding that Asian patients (mean: 5.2) had significantly better (lower) scores than those of either White (mean: 5.5) or Black (mean: 6.4) ethnicity.

Patients with a history of peripheral vascular disease had significantly worse (higher) VAQ scores (mean: 7.2 vs. 5.3, *p* = 0.011). Subgroup analysis (Additional file 1: Table S2) found that this difference in VAQ was largest in diabetic patients (mean: 8.1 vs. 4.4, *p* = 0.001), with no significant difference in VAQ by peripheral vascular disease in the non-diabetic cohort (mean: 6.0 vs. 5.8, *p* = 0.556).

There was no significant association with either cardiac disease (*p* = 0.055), diabetes (*p* = 0.195) or the overall duration of haemodialysis (*p* = 0.235). However, VAQ scores were found to differ significantly between dialysis units (*p* < 0.001), with means ranging from 3.6–7.8 (p < 0.001).

Associations between the VAQ score and factors related to the current access are assessed in Table 2. The score was found to vary significantly with the mode of vascular access (*p* = 0.004). Post-hoc analysis found patients with CVC scored significantly worse than those with AVF (mean: 6.6 vs. 5.1). Within each modality, no significant differences were detected between the anatomical locations of access. However, the VAQ was found to improve significantly with the duration of the current access (*p* = 0.003), from a mean of 8.9 at < 1 month, to 5.0 for those that were over a year. For the subset of patients with grafts or AVF, VAQ was not found to differ significantly between those where this was in the dominant vs. non-dominant arm (*p* = 0.341).

For patients with grafts and AVF, those requiring radiology intervention in the previous year had a significantly worse score (mean: 7.0 vs. 4.6, *p* < 0.001), although this trend was not observed in those with CVCs (*p* = 0.719, Table 3). Increasing numbers of previous CVC were associated with significantly worse VAQ scores, with means of 4.7 for those with no previous CVC, compared to 9.8 in those with > 10 CVC (*p* < 0.001).

Increasing satisfaction with the current access as well as its ease of use were associated with significantly better scores (all p < 0.001). Patients with a likelihood of recommending the current access also scored better (p < 0.001) (Table 4).

To identify independent predictors of the VAQ score a multivariable analysis was performed. The score was dichotomised based on the upper quartile, with VAQ > 7 treated as a “high” score (*N* = 203, 26.4%). The demographic factors from Table 5 were included in the model, the mode and duration of the current access, number of previous CVC and need for radiological interventions in the previous year. This model identified younger patient age (OR 0.70 per decade, 95% CI 0.61–0.79) and female gender (OR 2.23; 95% CI 1.55–3.22) to be the strongest independent predictors of a worse VAQ score. Scores were also significantly more likely to be worse in those with a history of cardiac disease (OR 1.69; 95% CI 1.13–2.52) and those requiring radiological interventions in the previous year (OR 1.75; 95% CI 1.16–2.63), and to vary significantly across the units (largest OR 6.12; CI 1.91–19.62). However, after accounting for these mode of current access was not found to be a significant independent predictor of VAQ scores (*p* = 0.767).

**Table 5:** Multivariable analysis of VAQ scores

	OR (95% CI)	*p*-Value
Age (per Decade)	0.70 (0.61–0.79)	**<0.001**
Gender (Female)	2.23 (1.55–3.22)	**<0.001**
Ethnicity		0.168
*White*	–	–
*Asian*	0.74 (0.44–1.25)	0.262
*Black*	1.39 (0.80–2.42)	0.246
*Mixed*	1.29 (0.09–18.16)	0.849
Peripheral Vascular Disease	1.44 (0.87–2.39)	0.158
Cardiac disease	1.69 (1.13–2.52)	**0.010**
Diabetes		0.482
*No*	–	–
*Diet Controlled*	0.59 (0.28–1.24)	0.162
*Tablet Controlled*	0.68 (0.27–1.70)	0.408
*Insulin*	0.95 (0.61–1.49)	0.829
Unit		**0.026**
*Unit 1*	–	–
*Unit 2*	1.82 (0.57–5.81)	0.313
*Unit 3*	2.08 (0.65–6.69)	0.218
*Unit 4*	2.95 (0.92–9.50)	0.069
*Unit 5*	2.92 (0.92–9.25)	0.069
*Unit 6*	1.97 (0.57–6.83)	0.285
*Unit 7*	2.31 (0.73–7.33)	0.154
*Unit 8*	3.60 (1.15–11.28)	**0.028**
*Unit 9*	4.96 (1.53–16.11)	**0.008**
*Unit 10*	6.12 (1.91–19.62)	**0.002**
Duration of Haemodialysis (per Year)	1.02 (0.98–1.06)	0.390
Current Access		0.767
*AVF*	–	–
*AV graft*	1.38 (0.58–3.25)	0.467
*Tunnelled CVC*	1.03 (0.62–1.71)	0.901
Duration of Current Access		0.124
*<1 month*	2.71 (0.93–7.88)	0.067
*1 month - 6 months*	1.25 (0.70–2.26)	0.450
*6 months − 12 months*	1.66 (0.97–2.87)	0.066
*over 1 year*	–	–
Radiology Intervention in the Last Year	1.75 (1.16–2.63)	**0.007**
Number of Previous CVC		0.474
*0*	–	–
*1 to 5*	0.93 (0.60–1.42)	0.725
*6 to 10*	1.63 (0.72–3.67)	0.239
*>10*	0.89 (0.28–2.87)	0.847

Results are from a multivariable binary logistic regression model, with a VAQ score > 7 as the dependent variable. The *N* = 2 patients with both CVCs and AVF were excluded, as were *N* = 4 with missing data on one of the factors, leaving *N* = 743 (*N* = 196 outcomes) for analysis. Odds ratios are relative to the reference category, or are for an increase of the stated number of units for continuous variables. Bold *p*-values are significant at *p* < 0.05

### Components of the VAQ score

**Fig. 1:**
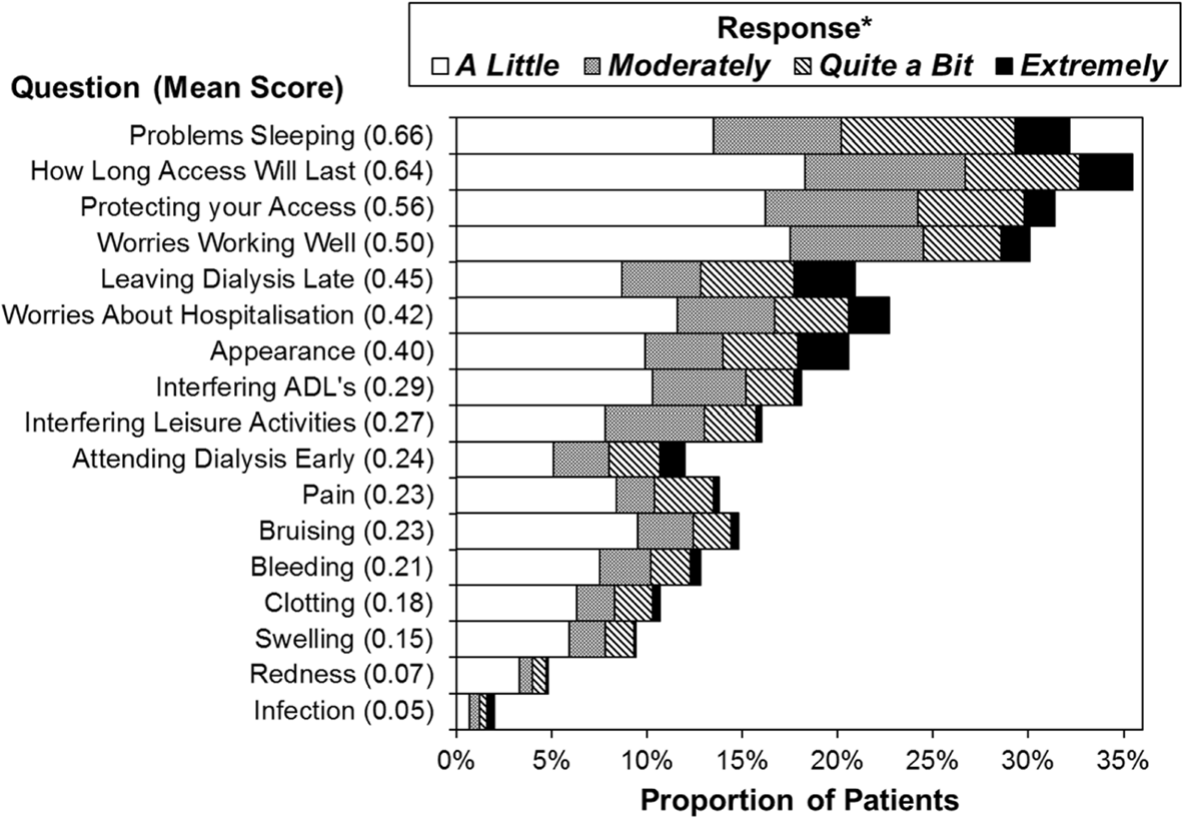
The questions related to how much patients had been bothered by the stated concern within the previous 4 weeks. The text of the questions has been abbreviated, with the full detail of the questions reported in Additional file 1: Table S1. Questions are sorted in descending order of the mean score, which is reported in brackets.*Patients responding “Not at All” were not included in the plot, but were considered when calculating the average scores

The VAQ score was broken down into its component questions, and analysed. The components with the best average scores were: infection and redness, with means of 0.05 and 0.07 respectively (Fig. 1). The worst average scores were for problems sleeping and worries over how long the access would last, having an average of 0.66 and 0.64 respectively.

The average scores for each question were compared across a range of demographic and access-related factors (Tables 6 and 7). The overall VAQ score had been shown to improve significantly with patient age. No significant associations were detected between patient age and the majority of the symptom-related questions. Scores for clotting were found to improve significantly with age (*p* < 0.001). Appearance became significantly less important with age, declining from a mean of 0.63 in < 55 years, to 0.23 in those aged 75+ (p < 0.001). Infection scores improved with increasing age, from a mean of 0.10 in the < 55 to 0.02 in the 75+ (*p* = 0.050).

**Table 6:** Comparisons of VAQ score components by demographic and access-related factors (part 1)

	Mean Score for the VAQ Question Relating to:							
	*Bleeding*	*Pain*	*Bruising*	*Swelling*	*Redness*	*Infection*	*Clotting*	*Appearance*
Age (Years)	*p = 0.071*	*p = 0.283*	*p = 0.232*	*p = 0.921*	*p = 0.328*	***p = 0.050***	***p < 0.001***	***p < 0.001***
*<55*	0.17	0.24	0.19	0.14	0.12	0.10	0.27	0.63
*55–64*	0.21	0.31	0.26	0.17	0.07	0.04	0.24	0.40
*65–74*	0.19	0.16	0.22	0.13	0.04	0.03	0.16	0.40
*75+*	0.27	0.20	0.24	0.14	0.06	0.02	0.06	0.23
Gender	*p = 0.575*	***p = 0.006***	***p = 0.005***	*p = 0.907*	*p = 0.692*	*p = 0.874*	***p < 0.001***	***p < 0.001***
*Female*	0.23	0.30	0.28	0.15	0.07	0.05	0.27	0.60
*Male*	0.20	0.17	0.19	0.14	0.07	0.04	0.11	0.26
Number of Previous CVC	*p = 0.655*	*p = 0.129*	*p = 0.510*	*p = 0.949*	*p = 0.078*	*p = 0.122*	***p < 0.001***	*p = 0.868*
*0–5*	0.22	0.21	0.23	0.14	0.06	0.03	0.15	0.40
*6+*	0.19	0.39	0.19	0.18	0.24	0.16	0.45	0.42
Current Mode of Vascular Access*	***p < 0.001***	*p = 0.068*	***p < 0.001***	***p < 0.001***	***p < 0.001***	***p < 0.001***	***p = 0.008***	*p = 0.392*
*AVF*	0.26	0.27	0.30	0.17	0.03	0.01	0.14	0.39
*AV graft*	0.24	0.18	0.21	0.29	0.00	0.06	0.32	0.21
*Tunnelled CVC*	0.06	0.12	0.02	0.06	0.21	0.17	0.26	0.46
Current Fistula**	*p = 0.257*	*p = 0.080*	*p = 0.831*	*p = 0.975*	*p = 0.921*	*p = 0.593*	*p = 0.172*	*p = 0.288*
*Brachiobasilic*	0.36	0.19	0.23	0.13	0.04	0.00	0.06	0.49
*Brachiocephalic*	0.32	0.33	0.33	0.18	0.03	0.00	0.17	0.44
*Radiocephalic*	0.17	0.19	0.29	0.15	0.03	0.01	0.14	0.33
Fistula/Graft on Dominant Arm**	*p = 0.136*	*p = 0.410*	*p = 0.638*	*p = 0.730*	*p = 0.850*	*p = 0.358*	*p = 0.567*	*p = 0.634*
*No*	0.25	0.27	0.28	0.17	0.03	0.01	0.16	0.41
*Yes*	0.28	0.23	0.34	0.15	0.02	0.00	0.14	0.33
Does Fistula/Graft on Dominant Arm Cause Issues**	*p = 0.516*	***p = 0.045***	***p = 0.016***	*p = 0.599*	*p = 0.258*	*p = 1.000*	*p = 0.254*	***p = 0.001***
*No*	0.28	0.14	0.22	0.16	0.03	0.00	0.10	0.20
*Yes*	0.27	0.46	0.65	0.14	0.00	0.00	0.22	0.62

*p*-Values are from Mann-Whitney or Kruskal-Wallis tests for comparisons across factors with two or three categories, respectively. For patient age, the exact value was correlated with the scores using Spearman’s rho. Bold p-values are significant at *p* < 0.05

*Excludes *N* = 2 patients with both CVC and AVF. **For patients where this question was applicable

**Table 7:** Comparisons of VAQ score components by demographic and access-related factors (part 2)

	Mean Score for the VAQ Question Relating to:								
	*Worries Working Well*	*Attending Dialysis Early*	*Leaving Dialysis Late*	*Problems Sleeping*	*Protecting Access*	*Interfering ADL’s*	*Interfering Leisure Activities*	*Worries About Hospitalisation*	*How Long Access Will Last*
Age (Years)	***p < 0.001***	*p = 0.149*	*p = 0.556*	***p < 0.001***	***p < 0.001***	***p < 0.001***	***p < 0.001***	***p = 0.003***	***p < 0.001***
*<55*	0.71	0.28	0.51	0.90	0.94	0.55	0.57	0.50	0.85
*55–64*	0.60	0.26	0.42	0.74	0.63	0.29	0.26	0.53	0.80
*65–74*	0.44	0.28	0.43	0.60	0.40	0.25	0.20	0.41	0.56
*75+*	0.29	0.16	0.43	0.44	0.31	0.12	0.10	0.27	0.41
Gender	*p = 0.116*	***p = 0.047***	***p = 0.037***	***p < 0.001***	*p = 0.214*	*p = 0.190*	*p = 0.520*	***p < 0.001***	***p = 0.004***
*Female*	0.56	0.28	0.52	0.80	0.63	0.36	0.30	0.54	0.77
*Male*	0.45	0.21	0.39	0.56	0.50	0.25	0.25	0.33	0.56
Number of Previous CVC	***p < 0.001***	*p = 0.413*	***p = 0.042***	***p = 0.035***	***p = 0.046***	*p = 0.089*	***p = 0.023***	***p < 0.001***	***p < 0.001***
*0–5*	0.45	0.23	0.42	0.63	0.53	0.28	0.25	0.35	0.56
*6+*	1.03	0.34	0.72	0.93	0.87	0.46	0.48	1.10	1.48
Current Mode of Vascular Access*	***p = 0.025***	*p = 0.370*	*p = 0.308*	*p = 0.635*	***p = 0.020***	***p < 0.001***	***p = 0.024***	***p < 0.001***	***p = 0.001***
*AVF*	0.43	0.21	0.41	0.64	0.50	0.22	0.23	0.33	0.54
*AV graft*	0.82	0.29	0.76	0.88	0.65	0.24	0.21	0.74	1.12
*Tunnelled CVC*	0.62	0.33	0.50	0.66	0.73	0.53	0.41	0.64	0.85
Current Fistula**	***p = 0.044***	*p = 0.988*	*p = 0.683*	***p = 0.032***	*p = 0.145*	*p = 0.217*	*p = 0.388*	*p = 0.467*	***p = 0.021***
*Brachiobasilic*	0.58	0.17	0.36	0.98	0.48	0.13	0.15	0.46	0.87
*Brachiocephalic*	0.37	0.21	0.45	0.67	0.43	0.22	0.22	0.34	0.53
*Radiocephalic*	0.48	0.23	0.36	0.54	0.58	0.24	0.26	0.27	0.48
Fistula/Graft on Dominant Arm**	*p = 0.936*	*p = 0.683*	*p = 0.957*	*p = 0.305*	*p = 0.621*	*p = 0.936*	*p = 0.303*	*p = 0.516*	*p = 0.556*
*No*	0.44	0.22	0.42	0.64	0.51	0.23	0.22	0.35	0.55
*Yes*	0.48	0.19	0.43	0.78	0.44	0.20	0.27	0.31	0.64
Does Fistula/Graft on Dominant Arm Cause Issues**	*p = 0.495*	*p = 0.063*	*p = 0.763*	*p = 0.801*	***p = 0.022***	***p < 0.001***	***p < 0.001***	*p = 0.937*	*p = 0.423*
*No*	0.44	0.26	0.44	0.73	0.32	0.03	0.09	0.31	0.56
*Yes*	0.57	0.03	0.41	0.89	0.73	0.59	0.70	0.32	0.81

*p*-Values are from Mann-Whitney or Kruskal-Wallis tests for comparisons across factors with two or three categories, respectively. For patient age, the exact value was correlated with the scores using Spearman’s rho. Bold p-values are significant at *p* < 0.05

*Excludes *N* = 2 patients with both CVC and AVF. **For patients where this question was applicable

Worries about the access working well, lasting, being protected and about requiring hospitalisation all declined significantly with age. Older patients scored significantly lower (showing less impact of the access) on the questions about having trouble sleeping, and interference with activities of daily living (ADLs) and leisure activities. Scores for attending dialysis early and leaving late did not differ significantly with age.

Analysis by gender found that females had significantly worse scores for pain, bruising and clotting than males. Females also scored significantly worse on the questions relating to appearance, problems sleeping, and worries about hospitalisation and how long the access would last. Concerns about attending dialysis early and leaving late were also significantly higher in females than in males.

Patients with six or more previous CVC had significantly worse scores on the clotting concerns component, as well as for problems sleeping, protecting their access and interference with leisure activities. These patients also scored significantly worse with regards to worries that the treatment is working well, how long the access will last, and about hospitalisation than those with fewer previous CVCs.

Comparisons across the modes of vascular access demonstrated significant differences for bleeding, bruising and swelling, for which the lowest scores were in CVCs. Significant differences in redness and infection were found, both of which were highest scores in CVC. A significant difference in the clotting scores was also detected, which were found to be lowest in AVF. Worries about the access working well, being protected, how long it would last and about hospitalisation were found to differ significantly by type of access, with average scores consistently being lower in AVF. Significant differences in the interference in ADLs and leisure activities were also observed, with scores being highest in tunnelled CVC.

For the subgroup of patients with AVFs, significant differences across types were detected for the questions relating to problems sleeping, concerns about how well the fistula was working and worries about how long the AVF would last. The scores for these were worse in Brachiobasilic but better in Radiocephalic AVF.

Comparisons between AVF and grafts in the dominant vs. non-dominant arm found no significant differences for any of the components. However, within the subgroup of patients with an AVF or graft in the dominant arm, those reporting that this caused issues scored considerably higher on the questions relating to interference with ADLs and leisure activities. In addition, these patients also had significantly higher scores for bruising, appearance, pain and for protecting access.

### Views on AVF vs. CVC by unit

Patients were asked which type of access they felt nurses preferred, and which they thought was best for their health. For the former, 36.3% of patients thought nurses preferred AVF and 9.5% responded with CVC, with 23.8% feeling that nurses were equally happy with two methods, and 30.4% being unsure. The majority of patients (73.9%) believed that AVF were better for their health, with 7.0% responding with CVC, 5.2% believing that there was no difference, and 13.9% unsure (Table 1).

**Fig. 2:**
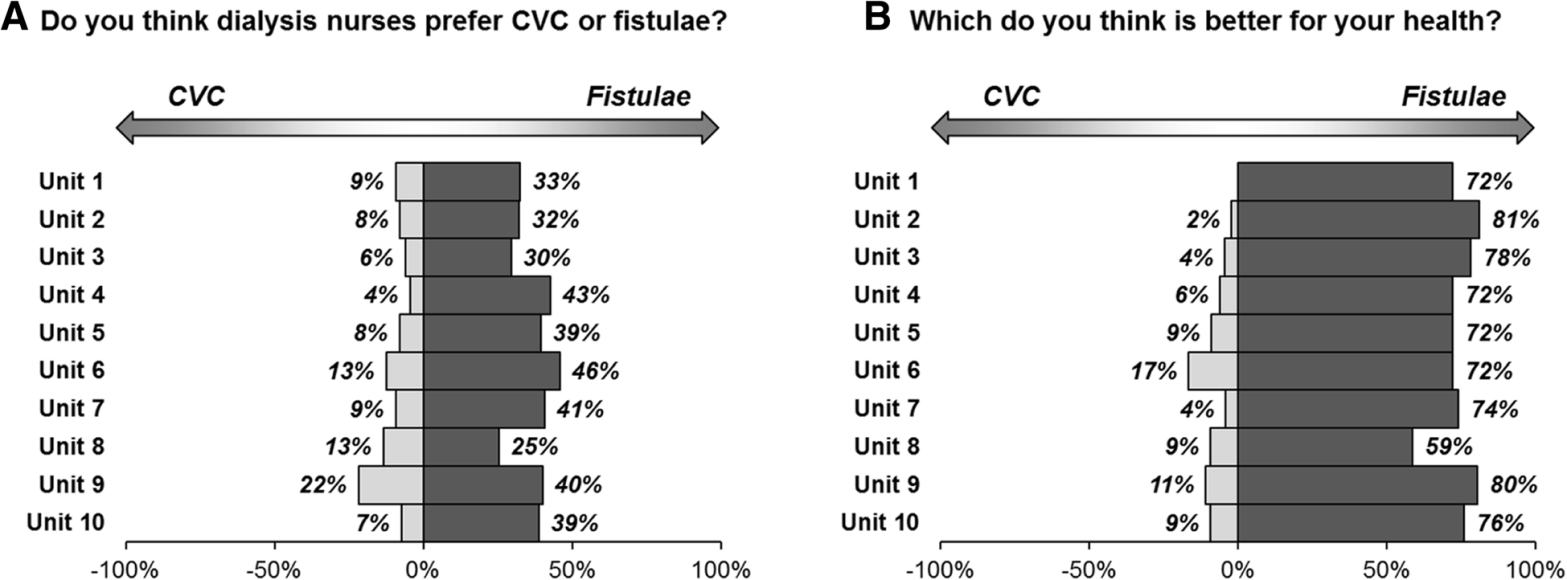
Thoughts on CVC vs. AVF by unit Plots represent the proportion of patients answering CVC or AVF – responses of “Equal” or “Not Sure” are not shown. The responses were converted into an ordinal variable, with categories of CVC, Equal/Not Sure combined, and AVF. This was then compared across units using a Kruskal-Wallis test, which returned p = 0.318 and p = 0.115 for questions A and B, respectively

These responses were then compared across the units. The responses of “Equal” or “Not Sure” were combined, and treated as a middle category, between CVC and AVF. Comparisons of the resulting variable found no significant differences in the responses to either question between units, with *p* = 0.318 and *p* = 0.115 respectively (Fig. 2).

### Free text analysis

Respondents dialysing on a CVC were asked whether they would consider changing to an AVF. For those who replied “no”, their reason was sought and categorised (Table 8). The majority of reasons related to concerns about physical (visual) implications of a fistula (34%) or because they were “happy on the line” (25%).

**Table 8:** Free text analysis

Reason	N (%)
Reasons for not considering fistula if current access is a CVC (*N* = 67)	
Concern about physical aspects of the fistula	
*Pain from the fistula*	12 (18%)
*Appearance of fistula*	5 (7%)
*Bleeding from the fistula*	4 (6%)
*Lifespan of a fistula*	1 (1%)
*Concerned about amount of maintenance required*	1 (1%)
Happy on a line/ feels a line is better for health	17 (25%)
Concern about having surgery	10 (15%)
Previous bad experience with fistula	7 (10%)
Surgical fatigue	7 (10%)
Holding out for a transplant	3 (4%)
Problems from fistula on dominant arm (*N* = 37)	
Poorer function of dominant hand overall	26 (70%)
Decreased function of dominant hand whilst on dialysis	10 (27%)
Pain	2 (5%)
Inability to self cannulate	1 (3%)
Is there anything else you wish you had known before starting? (*N* = 108^a^)	
More information in general/medical aspects dialysis	28 (26%)
More information on access choices:	
*In general*	13 (12%)
*Covering CVC as an option*	3 (3%)
More information about fistulas:	
*In general*	4 (4%)
*Bleeding risk*	2 (2%)
*Failure to mature risk*	1 (1%)
*Risk of steal*	1 (1%)
*Appearance*	8 (7%)
More information on mechanisms of dialysis	
*Difficulty in needling*	2 (2%)
*Need for needle rotation*	2 (2%)
*Size of needles*	1 (1%)
*Pain relating to cannulation*	10 (9%)
More information on renal replacement choices	11 (10%)
Information regarding impact of access on Activities daily living	7 (6%)
Crash landed so different info needed/discussed	6 (6%)
The amount of nephrology input would decrease on dialysis	3 (3%)
Discussion about the finite nature of vascular access options	2 (2%)
Need for intervention to maintain access	1 (1%)
Procedure for CVC removal	1 (1%)
More peer education	1 (1%)
Information on timing of access placement	1 (1%)

^a^Analysis based on the 108 responses who did want more information

To ascertain whether the presence of an AVF on the dominant arm caused a problem, this sub-group of patients were identified and analysed. Of the 125 patients with fistulae on the dominant arm, 37 (29.6%) reported that this was a problem. Of these, the majority (70%) felt overall hand function was poorer in their dominant arm, whilst 27% reported decreased function of the dominant hand whilst on dialysis (Table 8).

As part of service evaluation and improvement, all participants were asked whether there was anything they wished they had known before starting on dialysis. One hundred eight patients identified areas where they would have benefited from further information (Table 8).

## Discussion

Structured interviews has been shown to produce higher response rates and quality data capture than self-filled questionnaire studies and was hence employed [14, 15] and response rates were good with patients engaged in discussing their vascular access. Whilst only a comparatively small number of the population were not captured this must be acknowledged as a potential source of bias. Self-filled questionnaires have been suggested to provide freedom of thinking time and improve the accuracy of patient responses [16] however, we chose to replicate the method undertaken in the original VAQ and this could be identified as a potential weakness.

The VAQ was developed and validated in a Canadian haemodialysis population [12]. The VAQ indicated that patients are more satisfied with their access with increasing patient age and increasing age of their access; suggesting that there may be a period of adjustment whilst patients “get accustomed to” their access. This may be pertinent for counselling of patients prior to starting their access journey. For those patients who required intervention(s) to maintain their access, worse overall scores were seen. This may be lower levels of satisfaction, or could reflect the need for intervention heightening the patient’s awareness that the access may have a limited lifespan, increasing concern about future need for intervention.

Overall scores show better satisfaction with AVF than other modalities of access, although this was not found to be an independent predictive of satisfaction on multivariable analysis. When considering the components of the VAQ score separately, CVC’s showed greater impact on activities of daily living and leisure activities than other access modalities [5, 17]. As has been shown in other studies, the scores for the physical aspects (such as bleeding and appearance) of access were higher for AVFs, suggesting this maybe the reasoning behind the preference for CVCs [11, 17, 18].

From the multivariable analysis younger age and being female were both identified to give worse scores. These groups may be more conscious of their access and perceive its negative impact partly as a surrogate for the impact of their renal failure itself. As such a vital component of a patients ability to dialyse it may be that putting increased emphasis on these groups in the pre-dialysis counselling stages may be beneficial.

The presence of a fistula in the dominant arm did not seem to be of concern for the majority of patients. In those who did report issues, the VAQ score indicated that these largely related to appearance and interference with daily/leisure activities. Since vessel size in the dominant arm may be more favourable for fistula maturation, the longstanding dogma of avoiding the dominant arm should be challenged in selected patients, in favour of better vessel size [19].

Pain scores were not found to differ significantly between the different fistula types. Brachiobasilic fistulas have been suggested to be more painful (chronic pain and pain on cannulation) than brachiocephalic fistulas, but this has not been demonstrated in this cohort [20].

The VAQ differed significantly between dialysis units prompting further study to identify the causes and areas of the service in those units with the highest scores and least patient satisfaction to drive quality improvement. The difference between the scores in the units was not explained by different demographic differences between the units.

Differences, although not statistically significant, were identified between different dialysis units in terms of the patients perception of the nurse’s preference for dialysis access. Little research exists on the effect of the perception of the nurse’s preference on dialysis access modality choice, however, if patients feel that the nurses prefer CVCs then they might be more reluctant to consider changing from a CVC to a fistula. It also appears to be an area that would be sensible to target intervention and education towards.

Despite their access modality or preference, the majority of patients do seem to accept that fistulas are better for their health. However, despite this, the potential benefits do not outweigh the concerns about the physical aspects relating to the fistula. This is a finding that has been similarly highlighted in other research [11].

## Conclusion

Overall, patients are satisfied with their access and are acutely aware of the critical role it plays in their renal disease management. The ability to measure modifiable factors such as nurse attitude and centre effect may allow a unique approach to application of quality improvement initiatives and their outcomes.

## Additional file

Additional file 1: **Table S1.** Vascular Access Score questionnaire. **Table S2.** Associations between peripheral vascular disease and VAQ scores by diabetes status. (DOCX 16 kb)

## Data Availability

The raw data and materials are not available. This was not discussed with patients and this is not a research study it is part of a quality improvement project.

## Abbreviations

ADL: Activity of daily living
AVF: Arteriovenous fistula
AVG: Arteriovenous graft
CVC: Central venous catheter
HRQL: Health related quality of life
IQR: Interquartile range
OR: Odds ratio
QoL: Quality of life
VA: Vascular Access
VAQ: Vascular Access Questionnaire
