# Supplementary material for: The vascular access questionnaire: a single centre UK experience
Source: BMC Nephrol. 2019 Aug 5;20:299. doi: 10.1186/s12882-019-1493-9 (PMC6683579; doi:10.1186/s12882-019-1493-9)
Supplement: Supplementary file 1 — Table S1. Vascular Access Score questionnaire. Table S2. Associations between peripheral vascular disease and VAQ scores by diabetes status. (DOCX 16 kb) [file 12882_2019_1493_MOESM1_ESM.docx]

***Additional file 1: Table S1 Vascular Access Score questionnaire***

| ***Please rate on the scale how much in the last 4 weeks***  ***you have been bothered by:*** | **Not at all** | **A little** | **Moderately** | **Quite a bit** | | **Extremely** |
| --- | --- | --- | --- | --- | --- | --- |
| *Pain* |  |  |  | |  |  |
| *Bleeding* |  |  |  | |  |  |
| *Swelling* |  |  |  | |  |  |
| *Bruising* |  |  |  | |  |  |
| *Redness* |  |  |  | |  |  |
| *Infection* |  |  |  | |  |  |
| *Clotting* |  |  |  | |  |  |
| *Appearance* |  |  |  | |  |  |
| *Worries about the access not working well* |  |  |  | |  |  |
| *Attending dialysis early because of problems with the access* |  |  |  | |  |  |
| *Leaving dialysis late because of access problems* |  |  |  | |  |  |
| *Problems sleeping* |  |  |  | |  |  |
| *Concerns about protecting your access* |  |  |  | |  |  |
| *Access interfering with daily activities* |  |  |  | |  |  |
| *Access interfering with leisure activities* |  |  |  | |  |  |
| *Worries about needing to go to hospital because of your access* |  |  |  | |  |  |
| *Worries about how long your access will last* |  |  |  | |  |  |

***Additional file 1: Table S2 – Associations between peripheral vascular disease and VAQ scores by diabetes status***

|  | **Diabetic (N=299)** | | | **Non-Diabetic (N=450)** | | |
| --- | --- | --- | --- | --- | --- | --- |
|  | ***Mean*** | ***Median (IQR)*** | ***p-Value*** | ***Mean*** | ***Median (IQR)*** | ***p-Value*** |
| Peripheral Vascular Disease |  |  | **0.001** |  |  | 0.556 |
| *No* | 4.4 | 3 (1 – 7) |  | 5.8 | 3 (1 – 8) |  |
| *Yes* | 8.1 | 6 (2 – 10) |  | 6.0 | 4 (1 – 11) |  |

*p-Values are from Mann-Whitney tests and bold p-values are significant at p<0.05.*
